# Dual Drivers in the Myeloproliferative Neoplasms: An Underestimation?

**DOI:** 10.1177/15330338231179561

**Published:** 2023-06-13

**Authors:** Stephen E. Langabeer

**Affiliations:** 1 Cancer Molecular Diagnostics, Trinity College Dublin, St. James's Hospital, Dublin, Ireland

Dear Editor,

Recently published in *Technology in Cancer Research & Treatment*, Wang et al^1^ review the genetic and clinical characteristics of myeloproliferative neoplasms (MPN) with dual driver mutations suggesting that such patients have suboptimal responses with conventional treatments that require more customized therapeutic approaches. The incidence of such cases may be significantly underestimated in this review and can be largely attributed to the mutation detection methodology employed.

Several techniques are available for the routine detection of MPN driver mutations and include Sanger sequencing, allele-specific PCR, high-resolution melt analysis, fragment length analysis, and next-generation sequencing (NGS), each with its own methodological limitations of sensitivity and specificity and also clinical applicability. In the last few years NGS has become increasingly employed in routine molecular diagnostic laboratories and while lacking in sensitivity to targeted allele-specific real-time polymerase chain reaction, has the advantage of detecting (in addition to the classic driver mutations considered by Wang et al) single nucleotide polymorphisms (SNPs), benign variants and a considerable number of noncanonical mutations in these driver genes. While bioinformatics pipelines are able to filter out SNPs and benign variants, each noncanonical mutation requires either in silico or in vitro functional characterization to affirm pathogenicity.^2^ Co-existence of classical and functional, noncanonical driver mutations of the same gene have been frequently reported in *JAK2* exon 14, *CALR* exon 9, and *MPL* exon 10.^3‐5^ Even dual noncanonical mutations have been described.^6^

Given the increasing evidence that MPN patients with concurrent driver mutations have an inferior clinical outcome, detection of the second noncanonical (or canonical) mutation now appears entirely warranted for appropriate treatment decisions.^7^ It must also be acknowledged that the presence of a particular driver mutation, alone or in combination with another, does not entirely account for the clinical course of MPN patients: the germline host makeup, bone marrow micro-environmental factors, and acquisition of further somatic mutations in myeloid malignancy-associated genes can be responsible for modifying disease progression and outcome. Biologically, it is currently unknown whether if each driver mutation exists in a single or distinct clone impacts on clinical outcomes. Single-cell sequencing, a new approach not widely adopted in routine molecular diagnostics, has demonstrated further molecular complexity of MPN including intralineage clonal evolution.^8^

Coincident with this increasing appreciation of the dual driver MPN entity is the consequence of diagnostic algorithms and mutation detection technologies. The major time and cost benefits of NGS are the ability to sequence multiple targets (be they known mutational hot-spots or entire exon coverage) simultaneously on a unified technological platform therefore considerably reducing the requirement for a diagnostic algorithm that has multiple steps involving different methodological platforms.
